# Safety and efficacy of brivaracetam in children epilepsy: a systematic review and meta-analysis

**DOI:** 10.3389/fneur.2023.1170780

**Published:** 2023-07-06

**Authors:** Ting Song, Lingjun Feng, Yulei Xia, Meng Pang, Jianhong Geng, Xiaojun Zhang, Yanqiang Wang

**Affiliations:** ^1^Department of Neurology II, Affiliated Hospital of Weifang Medical University, Weifang, China; ^2^Surgical Department, Affiliated Hospital of Weifang Medical University, Weifang, China

**Keywords:** brivaracetam, children epilepsy, seizures, the retention rate, adverse events

## Abstract

**Background:**

Epilepsy is one of the most common neurological diseases, affecting people of any age. Although the treatments of epilepsy are more and more diverse, the uncertainty regarding efficacy and adverse events still exists, especially in the control of childhood epilepsy.

**Methods:**

We performed a systematic review and meta- analysis following the Cochrane Handbook and preferred reporting items for systematic reviews and meta-analyses (PRISMA) guidelines. Four databases including PubMed, Embase, Web of Science and Cochrane library were searched. Studies reporting the use of brivaracetam monotherapy or adjuvant therapy in children (aged ≤18 years) were eligible for inclusion. Each stage of the review was conducted by two authors independently. Random-effects models were used to combine effect sizes for the estimation of efficacy and safety.

**Results:**

A total of 1884 articles were retrieved, and finally 9 articles were included, enrolling 503 children with epilepsy. The retention rate of BRV treatment was 78% (95% CI: 0.64–0.91), the responder rate (reduction of seizure frequency ≥ 50%) was 35% (95% CI: 0.24–0.47), the freedom seizure rate (no seizure) was 18% (95% CI: 0.10–0.25), and the incidence rate of any treatment-emergent adverse events (TEAE) was 39% (95% CI: 0.09–0.68). The most common TEAE was somnolence, which had an incidence rate of 9% (95% CI: 0.07–0.12). And the incidence rate of mental or behavioral disorders was 12% (95% CI: 0.06–0.17).

**Conclusion:**

Our systematic review and meta-analysis showed that BRV seemed to be safe and effective in the treatment of childhood epilepsy.

## 1. Introduction

Epilepsy is a common pediatric neurological disease, and the prevalence of epilepsy in children is 0.5–1% (1). The sequelae of childhood epilepsy including mental disorders and behavioral problems will have a negative impact on adults’ education and employment (2, 3). Therefore, the social outcome of epilepsy set in childhood period is worse than that of contemporary adults’ epilepsy. Controlling epilepsy in childhood, and effectively achieving remission of epilepsy can greatly improve social outcomes and reduce economic burden (1, 3). However, the existence of various types of childhood epilepsy, poor compliance, long treatment time, adverse reactions, complications and other factors make the diagnosis, treatment and related clinical trials of pediatric epilepsy more difficult. Treatments for epilepsy include antiseizure medication (ASM), epilepsy surgery, vagus nerve stimulation, ketogenic diet, as well as targeted therapy. Whereas, due to the particularity of children, the safety and tolerance of these treatments have great limitations, so ASM is still the main treatment (4). At present, the number of auxiliary ASMs [including topiramate (5), levetiracetam (LEV) (6, 7), oxcarbazepine (OXC) (8, 9), zonisamide (10), etc.] that have completed randomized, placebo-controlled trials for childhood epilepsy is limited. The sensitivity and tolerance of children with epilepsy to these ASMs is decreasing, and the control of epilepsy is becoming insufficient. It is high time to introduce new antiepileptic drugs to control children epilepsy efficiently.

Brivaracetam (BRV), a new antiepileptic drug, is a highly selective ligand of synaptic vesicle protein 2A (SV2A), which has a structure similar to levetiracetam (LEV). It was reported that the affinity of BRV for SV2A is about 15–30 times that of LEV (11). In 2016, BRV was approved by the FDA and EMA to assist in the treatment of focal epilepsy in adults (≥16 years old) (12). In 2018, the United States approved epilepsy patients aged ≥4 years as the applicable population for BRV as adjunctive therapy and monotherapy (13). Pre-clinical studies and clinical data reported that BRV can be made into conventional tablets and oral liquids easily accepted by children. Given that BRV has lipid solubility, it can swiftly cross the blood–brain barrier and subsequently cause a rapid onset by occupying SV2A *via* intravenous administration, making it an attractive medicine for emergencies such as status epilepticus (11, 14, 15). Additionally, BRV may be effective for focal epilepsy, generalized epilepsy and epilepsy syndrome (11).

Clinical studies on the efficacy and safety of BRV have been carried out, especially in adults with epilepsy. Relevant adults’ data and results can be extrapolated to childhood epilepsy. Manuel (16) has summarized phase IIb (NCT00175929, NCT00175825), phase III/III b (NCT00490035, NCT00464269, NCT00504881, and NCT01261325), LFFU (NCT00175916, NCT00150800, and NCT01339559) trials involving a total of 2,186 adults with partial-onset seizures (POS) that received adjuvant BRV (50–200 mg/d) treatment. These trials reported that the retention rate at 6, 12, 24 months was 91, 79.8, and 68.1% respectively, and the freedom seizure rate was 4.9, 4.2, and 3.0%, respectively. The median reduction of POS frequency during the treatment period was 48.8%, and the response rate was 48.7%. It was also reported that TEAE incidence rate was 84.5%. These results indicate that the use of adjuvant BRV in adult epilepsy has good long-term safety and efficacy. A retrospective cohort study in 2018 reported the use of BRV’s acute intravenous treatment for hereditary generalized epilepsy (GGE) and status epilepticus (SE). The 3-month retention rate was 82.4%, and the response rate was 36%. 26% of patients had somnolence, ataxia, and psychological or behavioral disorders, and the adverse behavioral events were lower than LEV. Therefore, it was considered feasible to convert from LEV to BRV immediately, and to provide a reference for childhood epilepsy (17). Pellock believed that the efficacy of ASM on POS could be extrapolated from adults to children (over 2 years old) through clinical evidence, such as neurophysiology (EEG) and electrophysiology of clinical behavior evolution (18). Schoemaker extrapolated the exposure response model of adults with POS, treated with BRV, to children, and determined the pharmacokinetic characteristics of BRV for children with epilepsy, and the maximum response dose for children (over 4 years old) was 4 mg/kg/d (19). In the above adult epilepsy trials and several epilepsy animal model experiments, it has been confirmed that the conversion from LEV to BRV, or auxiliary BRV treatment, maintains at least the same level of seizure control and has lower behavioral adverse reaction rate than LEV (11, 20, 21). In addition, FDA also recognizes the efficacy inference of ASM from adults to children (15). Although these evidences can play a reference role in BRV treatment of childhood epilepsy, unfortunately, there is no relevant clinical trial and direct evidence, let alone a meta-analysis about it. Therefore, further data and consideration are needed urgently.

The purpose of our systematic review and meta-analysis is to analyze relevant studies, to explore the efficacy and safety of BRV in children with epilepsy. This study is the first one to conduct a meta-analysis of all literature involving the use of BRV for children with all types of seizures, aiming to verify the outcomes of BRV in children. We hope that the finding of the review will provide a reference for the clinical application for children epilepsy.

## 2. Methods

### 2.1. Literature search strategy

This systematic review and meta-analysis had been prospectively registered in PROSPERO under the registration number of CRD42022368200. Two independent reviewers searched the English literature from PubMed, Embase, Web of science and Cochrane until October 2022 respectively, and conducted a systematic review and meta-analysis of the single-arm and observational retrospective studies. The retrieval was comprehensively conducted by combining mesh words “brivaracetam” and “epilepsy” with corresponding free words. See Supplementary Table S1 for the literature retrieval strategy of the electronic databases.

### 2.2. Inclusion and exclusion criteria

We implemented the emission standards of this study according to the following “PICOS” principles.

#### 2.2.1. Participants/population

Inclusion criteria included: (1) According to the international definition of children, the patients who were under 18 years old were eligible; (2) All types of epilepsy that met the diagnostic criteria of epilepsy of the International League Against Epilepsy (ILAE) (1).

The exclusion criteria was: (1) Patients>18 years old. (2) Epilepsy caused by brain tumor, progressive encephalopathy or other progressive neurodegenerative diseases.

#### 2.2.2. Interventions

Inclusion criteria: (1) All BRV studies, whether BRV was administered alone or as an adjunctive treatment combined with other ASM. (2) All administration methods of BRV were studied, regardless of tablet preparation, oral solution, intravenous drip and intravenous bolus injection.

#### 2.2.3. Types of studies to be included

Inclusion: (1) The literatures published in English were included, and the eligibility was not limit by country, gender or setting. (2) The follow-up time was sufficient to reflect the outcome indicators of the study.

Literature such as reviews, conference abstracts, non-English, unrelated case reports, animal experiments, literature that had inconformity of target population, literature that had unobtainable full text, and from which we were unable to extract effective outcome indicators were excluded (22).

### 2.3. Outcomes

The main outcome of a randomized controlled trials of adjuvant BRV treatment for adult epilepsy was the frequency of weekly POS seizures in the treatment group compared to the placebo group during treatment. However, the studies we included were single-arm trials and retrospective studies, with the retention rate as the primary measure, summarizing the efficacy and tolerability of ASM. The retention rate of our main results refers to the proportion of children who did not stop using BRV treatment due to poor efficacy or adverse reactions, which can reflect the efficacy and safety of BRV treatment for childhood epilepsy effectively. At the same time, we analyzed the proportion of children who withdrew from treatment due to TEAE and poor efficacy.

Secondary outcomes were responder rate (defined as the proportion of children whose seizure frequency was reduced by ≥50% compared with the baseline period), seizure freedom rate (the proportion of children who had no seizure compared with the number of seizures in the baseline period), the incidence of any TEAE after receiving BRV, and the incidence of the common adverse event (somnolence and mental/behavioral disorders) in children (23).

### 2.4. Data extraction

The data of each study included were extracted by two reviewers independently, and the disputed parts were determined through discussion with the third reviewer. The extracted data included first author, publication year, country, type of study design, intervention, dose, age, sample size, sex ratio, average age of initial seizure, follow-up time, and outcome indicators (such as the proportion of children who did not withdraw from the trial, the number of participants with positive reaction, epilepsy freedom, side effects, somnolence and mental/behavioral disorders). For missing data, we contacted the author to ensure the integrity of the data, if necessary.

### 2.5. Risk of bias and quality assessment

The quality assessment and bias risk assessment were conducted by two reviewers independently, and a third reviewer was consulted to solve the disagreements. Evidence-based medicine suggests using methodological index for non-randomized studies (MINORS) scale to evaluate the bias risk of single-arm researches. The first 8 items of research evaluation in the non-control group have a maximum score of 16 points, while 12 items in the control group have 24 points in total. Conventionally, studies with a score ≥ 12 points means that the quality is good and can be admitted into the analysis (24). The cross-sectional studies can be evaluated according to the Joanna Briggs Institute (JBI) manual, with a maximum score of 20 points. The study with a score of more than 14 points is of good quality (25, 26).

### 2.6. Statistical analysis and publication bias

The sample size data were analyzed by intention to treat (ITT). We adopted STATA (version 15.1) for statistical analysis and publication bias assessment. We visually checked the forest map to estimate the degree of heterogeneity, and used Cochran’s Q test *I*^2^ to assess the heterogeneity (27). *I*^2^ < 50% was considered as low heterogeneity, and fixed effects model was used. If *I*^2^ > 50% or *p* > 0.1, it was considered that there was significant heterogeneity, and a random effects model was used to evaluate the combined effect amount. For the secondary variables, the aggregate effect was described by the single rate (*p-*value) of 95% confidence interval (95% *CI*).

According to the Cochrane Intervention System Evaluation Manual, a funnel chart was drawn for at least 10 studies to check the symmetry (21)_._ Due to the small number of included articles (less than 10 articles), it was not suitable to draw a funnel chart to evaluate publication bias. Egger’s test was used to evaluate the publication bias quantitatively, and *p* > 0.05 indicated that there was no significant publication bias.

### 2.7. Subgroup

If the heterogeneity is significant and the number of literatures is sufficient, we plan on performing a subgroup analysis to assess the possible source of heterogeneity. Subgroup analysis would be conducted by different research types, country, age stratification, sex ratio, race, intervention drug dose and follow-up time, to reduce heterogeneity.

### 2.8. Sensitivity analyses

We conducted a sensitivity analysis to assess the stability of the outcome indicators included in the study.

## 3. Results

### 3.1. Study selection

**Figure 1:**
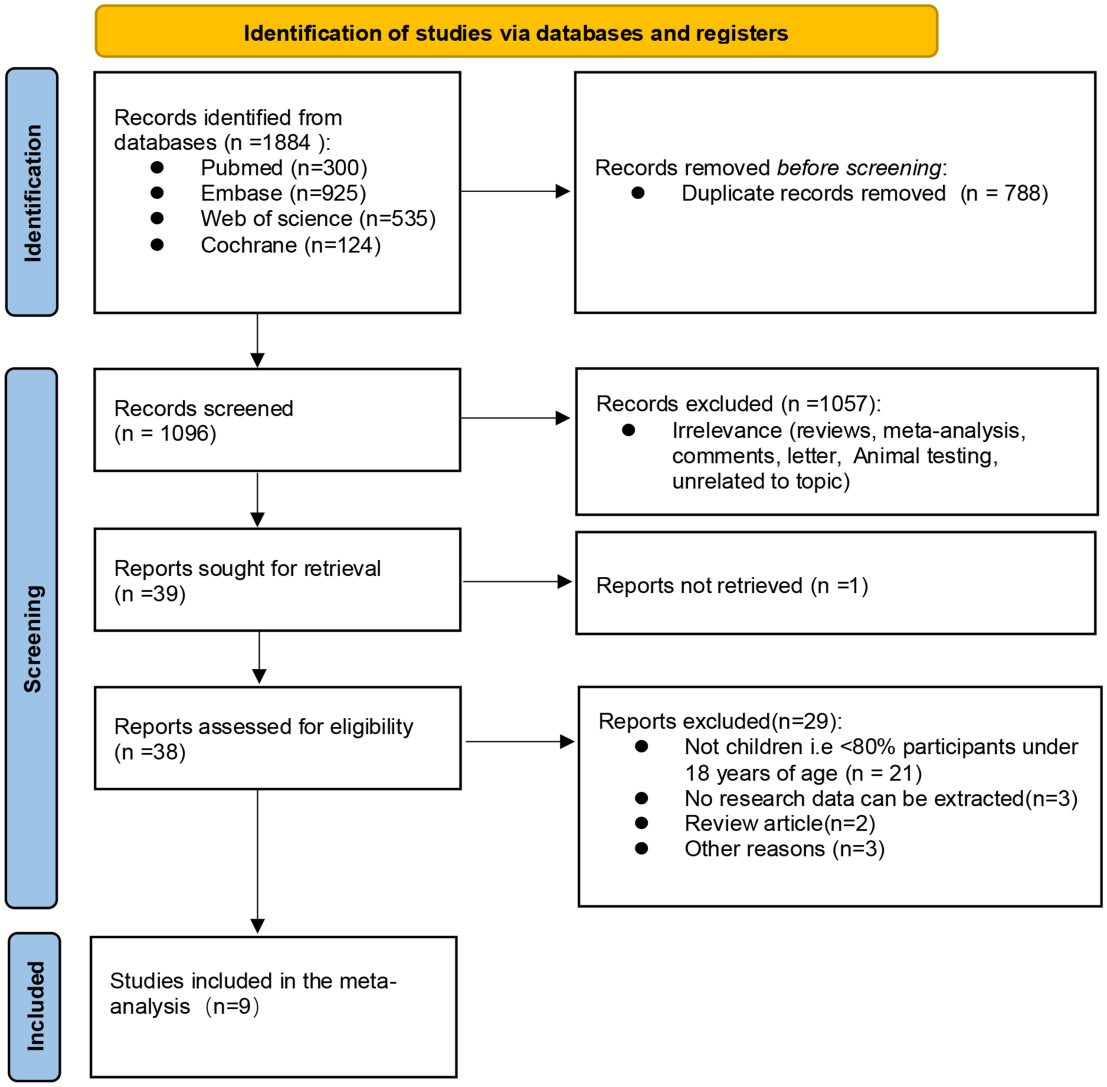
PRISMA (preferred reporting items for systematic reviews and meta-analyses) flow diagram, the final 9 studies were included after inclusion and exclusion criteria.

A total of 1884 documents were obtained through electronic database retrieval, and 1,096 documents remained after removing duplicate documents. Then, 1,057 documents were excluded by screening the titles and abstracts of the literatures and 39 full texts had to be reviewed. Thirty articles were excluded because they did not meet the inclusion criteria (1 article failed to obtain the full text, 21 articles did not have a target population, 3 articles had no extractable data, 2 were review articles, 2 articles for other reasons), and finally 9 articles were included (see Figure 1 for the selection process).

### 3.2. Study characteristics and assessments of risk of bias

This study includes eight single-arm studies (12, 13, 28–33) and one cross-sectional study (34). In these nine studies, there were four multicenter studies (carried out in several countries), two trials in two hospitals, and three single-center studies, including in total 503 children aged <18 years, with the proportion of men and women accounting for 52.1 and 47.9%, respectively. The study characteristics of the included literature, and the baseline characteristics of the participants, are shown in Table 1 and Supplementary Tables S2, S3. The quality evaluation of the single-arm studies was conducted according to the MINORS scale, while the cross-sectional study used the JBI scale to assess the risk of bias (see Tables 2, 3 for the bias risk scores of the included literatures), which indicated that nine studies could be included in this meta-analysis.

**Table 1:** Characteristics of included studies.

Study (author/year)	Country	Design	Treatment arms (*n*)	Dose (mg /kg/d)	Mean age (years)	SD	Range	No of patients	F/M	Mean age at epilepsy onset (years)	SD	Follow up (week)
28	Multicenter, 4 German epilepsy centers (Frankfurt am Main, Marburg, Münster, and Neuruppin)	Multicenter, retrospective study	BRV	104.0 ± 57.7 mg/d	12.2	4.2	3–17 years	34	19/15	7.1	4.5	12
29	Multicenter, 29 centers in the USA, Mexico, Belgium, Czech Republic, Poland, and Spain	Phase IIa, open-label, single-arm, fixed three-step dose escalation trial	BRV	3.2	6.3	4.8	1 month to 16 years	99	51/48	2.5	3.3	8
34	Safra Children Hospital, Sheba Medical Center, Israel	Cross-sectional retrospective chart review	BRV	3.8 ± 1.8	13.8	4.07	6.9–20 years	31	10/21	5.7	3.7	12
30	St. Christopher’s Hospital for Children in Philadelphia, Pennsylvania	Retrospective chart review	BRV	3.9	12.5	NA	4–16 years	20	14/6	NA	NA	Included 28
31	Multicenter, 29 centers in the USA, Mexico, Belgium, Czech Republic, Poland, and Spain	Phase IIa, open-label, single-arm, three-step dose-escalation trial	BRV	3.85	9.5	3.5	1 month to 16 years	149	65/84	5.6	3.6	192
32	2 Catalan university hospitals (Hospital Arnau de Vilanova, Lleida and Hospital Vall d’Hebron, Barcelona).	Observational and retrospective study	BRV	2.8	11	NA	<18 years	46	31/15	2.7	NA	48
12	Ni˜no Jesús Hospital in Madrid	Retrospective and descriptive study	BRV	4.3	8.8	NA	6-12 years	66	23/43	2.5	NA	12
33	IRCCS Neurological Science Institute of Bologna and IRCCS “Medea” La Nostra Famiglia of Conegliano	Retrospective study	BRV	3.5	11.9	2.5	8 years and 4 months to 14 years	8	4/4	0.58	NA	32
13	Multicenter,37 sites across 7 countries (Czech Republic, Germany, Hungary, Italy, Mexico, Spain, and the United States)	Phase 2, multicenter, open- label trial	BRV	1.1 ± 0.3	6.4	4.7	1 month to 16 years	50	24/26	2.6	3.4	10

BRV, brivaracetam; SD, standard deviation.

**Table 2:** Risk of bias assessment by MINORS scale.

ID	IF	First Author	Year	Study design	A clearly stated aim	Inclusion of consecutive patients	Prospective collection of data	Endpoints appropriate to the aim of the study	Unbiased assessment of the study endpoint	Follow-up period appropriate to the aim of the study	Loss to follow up less than 5%	Prospective calculation of the study size	An adequate control group	Contemporary groups	Baseline equivalence of groups	Adequate statistical analyses	Total score
Schubert-Bast (28)	3.337	Schubert-Bast	2018	retrospective study	2	2	2	2	0	2	2	0					12
Liu (29)	3.930	Liu	2019	single-arm	2	2	2	1	0	2	2	1					12
McGuire (30)	2.363	McGuire	2019	retrospective	2	2	2	2	0	2	2	0					12
Patel (31)	3.692	Patel	2019	single-arm	2	2	2	2	0	2	2	0					12
Visa-Reñé (32)	3.337	Visa-Reñé	2020	observational and retrospective study	2	2	2	2	0	2	2	0					12
Ferragut (12)	2.991	Ferragut	2021	retrospective and descriptive study	2	2	2	2	0	2	2	0					12
Russo (33)	JCI0.19	Russo	2021	retrospective study	2	2	2	2	0	2	2	0					12
Farkas (13)	6.740	Farkas	2022	Phase 2, multicenter, open- label trial, research article	2	2	2	2	0	2	2	0	2	2	2	1	19

The items are scored 0 (not reported), 1 (reported but inadequate) or 2 (reported and adequate). The global ideal score being 16 for non-comparative studies and 24 for comparative studies.

**Table 3:** Risk of bias assessment by JBI scale.

ID	First Author	Year	IF	Study design	Is the purpose of the study clear? Is the basis sufficient?	How is the study population selected (whether the subjects are randomly selected, and whether stratified sampling is adopted to improve the representativeness of the sample)?	Are the inclusion and exclusion criteria of the sample clearly described?	Are sample characteristics clearly described?	Are the data collection tools reliable and valid (e.g. Using investigator surveys, How repeatable are the findings)?	What are the measures to verify the authenticity of the information	Whether to consider ethical issues?	Is the statistical method correct?	Whether the presentation of the results of the study is appropriate and accurate (whether the results and inferences are distinguished, and whether the results are true to data rather than inferences)	Is the research value clearly explained?	Total Score
Nissenkorn (34)	Nissenkorn	2019	3.337	Cross-sectional retrospective chart review	2	0	1	2	1	0	2	2	2	2	14

Evaluation criteria: 0, not meeting the requirement; 1, mentioned, but not described in detail; 2, comprehensive and correct description.

### 3.3. Outcomes

#### 3.3.1. Primary outcomes

##### 3.3.1.1. Retention rate

**Figure 2:**
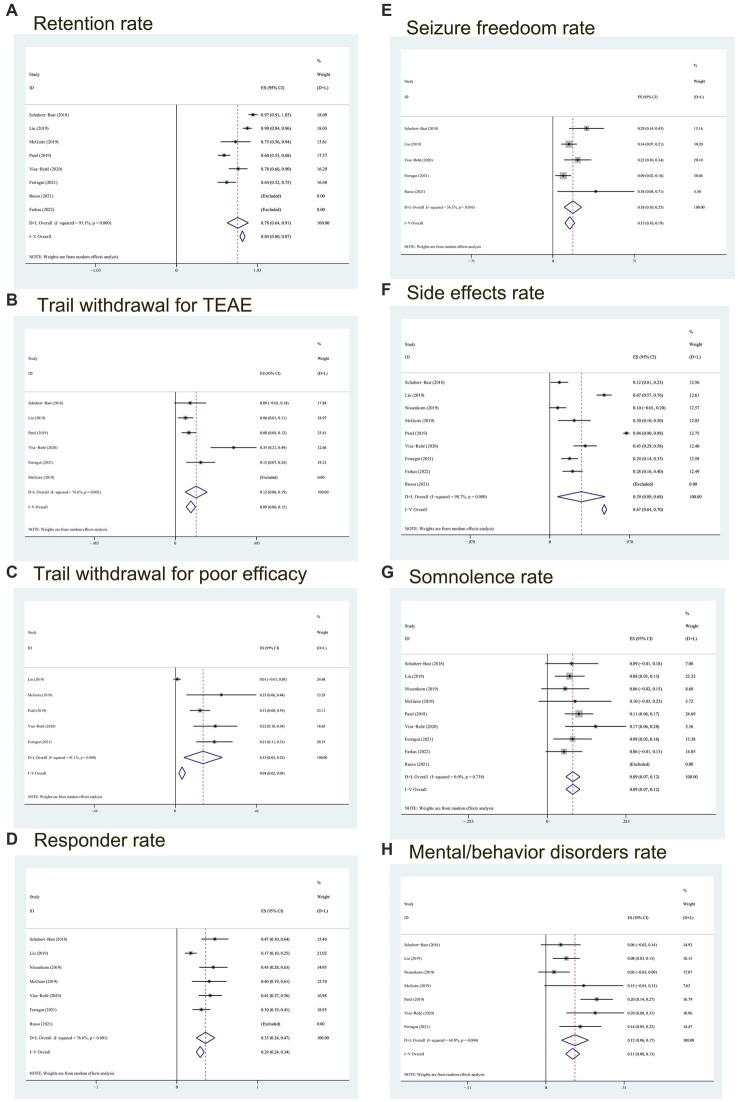
Forest plot of outcomes. **(A)** The retention rate; **(B)** trial withdrawal probability for TEAE; **(C)** probability of poor efficacy leading to discontinuation of the BRV; **(D)** the responder rate; **(E)** probability of seizure freedom; **(F)** the side effects rate; **(G)** the somnolence rate; **(H)** the mental/behavior disorders rate. CI, confidence interval; Heterogeneity (*I^2^*).

A meta-analysis was conducted on the retention rate of six articles in this study. The retention rate of 4 reports was>90%, of which 2 reports was 100%. After heterogeneity test, *I*^2^ = 93.1% and *p* < 0.05 of *Q*-test, indicating that there was significant heterogeneity between the selected literatures in this study. Based on the random effect model, the total effect amount was 0.78, and the 95% confidence interval was 0.64–0.91, which is statistically significant. The results suggest that the retention rate of BRV treatment for childhood epilepsy is 78% (see Figure 2A).

In addition, according to the analysis of the rate of withdrawal from treatment due to TEAE and poor efficacy, we used the random effects model to conclude that the proportion of withdrawal from treatment due to TEAE was 12% (95% *CI*:0.06–0.19) (see Figure 2B), and the quit rate because of poor efficacy was 15% (95% *CI*, 0.05–0.25) (see Figure 2C).

#### 3.3.2. Secondary outcomes

##### 3.3.2.1. Responder rate

We analyzed seven trials including 304 children. The meta-analysis results *I*^2^ = 76.6% (*p* < 0.05) showed that there was significant heterogeneity, and the responder rate was 35% (95% *CI*: 0.24–0.47) (see Figure 2D).

##### 3.3.2.2. Seizure freedom

5 studies reported on the proportion of children without seizures in the follow-up period compared with the baseline period, involving 47 children with freedom seizures during BRV treatment. Heterogeneity test results (*I*^2^ = 56.5%, *p* = 0.056) showed that the studies were of high heterogeneity resulting in the selection of the random effects model. And referring to Figure 2E, the total freedom seizure rate was 18% (95% *CI*: 0.10–0.25).

##### 3.3.2.3. Side effects

We analyzed the TEAE of BRV in the treatment of childhood epilepsy from two aspects: the incidence of any TEAE and the occurrence of the most common TEAE. Adverse reactions after BRV treatment include lethargy, loss of appetite, behavioral disorders (such as aggression, irritability, impulsion), etc. Among them, somnolence is the most common adverse event.

All 9 studies reported on the proportion of children with any adverse reactions, and the combined incidence was 39% (95% *CI*: 0.09–0.68, *p* = 0) according to the random effects model (see Figure 2F). It can be seen from Figure 2 that the occurrence of adverse reactions has a large heterogeneity (*I*^2^ = 98.7%), which may be caused by the combination of several ASMs during the observation/trial period, or using other ASMs before inclusion.

Eight studies enrolling 49 patients who suffer from somnolence reported that heterogeneity (with *I*^2^ = 0%, *p* = 0.738 > 0.1) (see Figure 2G) was low, so the analysis was conducted with the fixed effects model. It was observed that the combined incidence of somnolence was 0.09 (95% *CI*: 0.07–0.12). The results of this meta-analysis showed that 9% of children had somnolence.

Mental/behavioral adverse reactions are also common side effects of BRV. Neurobehavioral disorders, including behavioral adverse reactions (e.g., depression, aggression, irritability), psychosis comorbidity, and cognitive disturbance, were reported in 7 studies using BRV for epilepsy in children. The Figure 2H showed moderate heterogeneity (*I*^2^ = 68.6%, *p* = 0.004) between studies, so a random effect model was used. As can be seen from Figure 2H, the total effect size of mental or behavioral disorders was 0.12 (95% *CI*:0.06–0.17), which means that the incidence of neurobehavioral adverse reactions was 12%.

#### 3.3.3. Subgroup

Due to the insufficient number of articles reporting relevant indicators, there was only one article in the subgroups. Considering that reason, we did not carry out the proposed subgroup analysis.

### 3.4. Publication bias

The publication bias was evaluated by Egger’s test. The results of the Egger’s test, *p-*value, about retention rate, responder rate, seizure freedom rate, somnolence rate and the incidence rate of mental or behavioral disorders were *p* = 0.063, 0.563, 0.973, 0.777, 0.226 > 0.05 respectively, indicating no significant publication bias. The Egger’s test of side effect rate showed that the *p-*value was 0.001 < 0.05, indicating that there was a significant publication deviation. It may be that the study with insignificant statistical effect and insufficient sample size due to obvious withdrawal from BRV treatment was not published.

### 3.5. Sensitivity analyses

**Figure 3:**
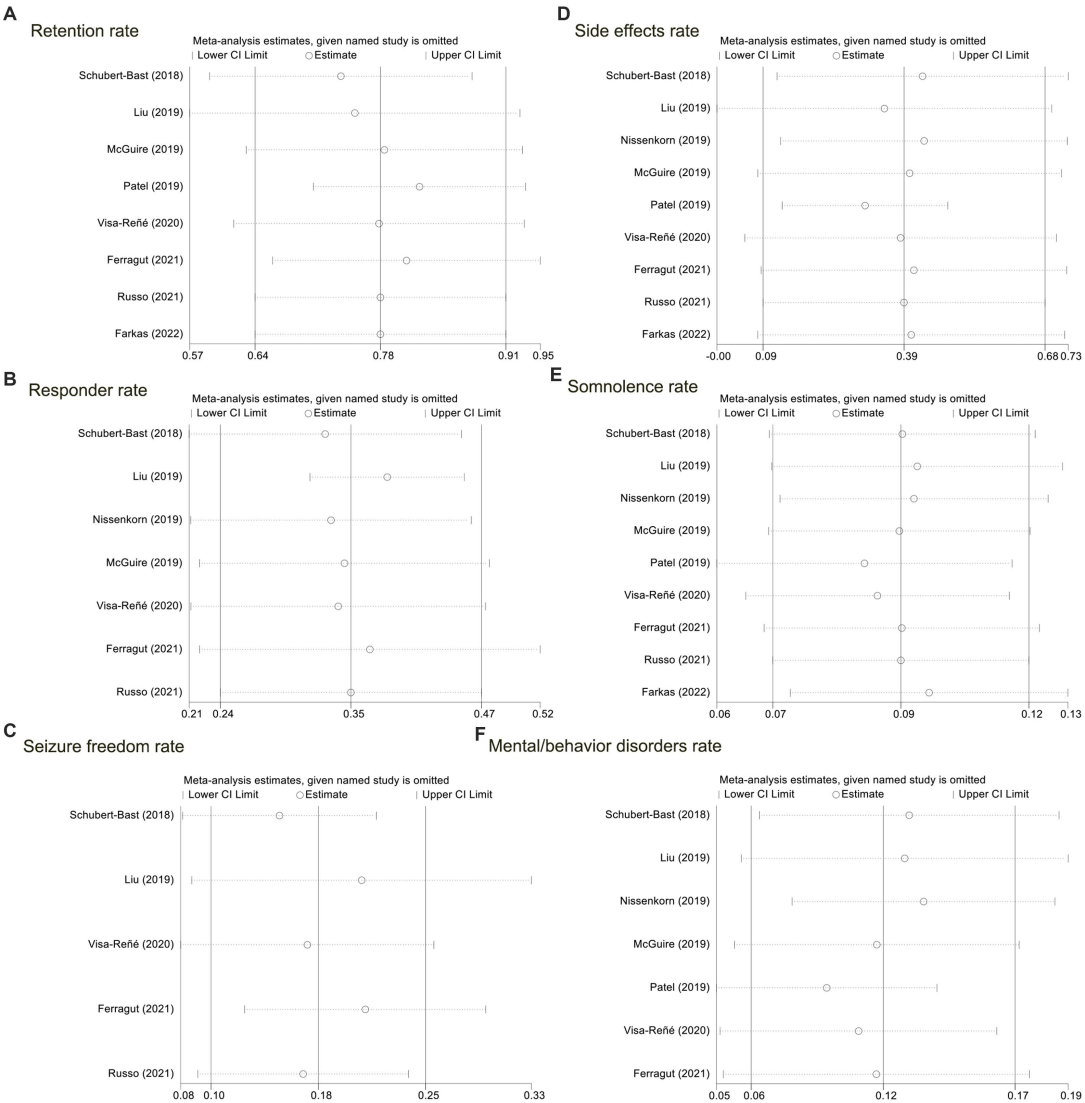
Sensitivity analyses for the nine included studies. **(A)** The retention rate; **(B)** the responder rate; **(C)** probability of seizure freedom; **(D)** the side effects rate; **(E)** the somnolence rate; **(F)** the mental/behavior disorders rate.

In our analysis, excluding any article did not affect the overall outcome of the article. Therefore, all sensitivity analyses associated with the meta-analysis performed in this study suggested stable results (please refer to Figure 3).

## 4. Discussion

Our systematic review and meta-analysis is the first to evaluate and analyze the safety and effectiveness of BRV, as a single drug or adjuvant drug, in the treatment of childhood epilepsy systematically. In our study, we analyzed nine articles including three open-label trials and six retrospective studies, in which 503 children were involved. The findings indicate that BRV is beneficial for the majority of types of epilepsy and is generally well tolerated and safe. The main findings of our study were that the retention rate of BRV treatment in childhood epilepsy is reasonable. There were three main reasons for withdrawal from BRV treatment: adverse reactions, poor efficacy, and patients’ willingness. Actually, in included studies, most patients terminated the treatment due to adverse reactions and insignificant efficacy. Therefore, the retention rate of BRV is a summary of ASM’s feature, which can comprehensively assess safety and efficacy relatively. This meta-analysis showed that the retention rate of children participating in the BRV treatment trial was 78%, which is consistent with the retention rate at 12 months of the adult trial in Manuel’s study (14). In the cases of drug withdrawal, adverse reactions (12%) and poor efficacy (15%) accounted for the majority.

The long-term prognosis of childhood epilepsy depends on remission, recurrence, seizure freedom, neurological impairment, medication, etc. (35). So far, a large number of clinical trials have reported effective response rate of BRV in the treatment of adult epilepsy. A phase IIb study (N01193; NCT00175825) that enrolled adult patients with refractory POS treated by BRV showed that the responder rates in the 5 mg/d, 20 mg/d, 50 mg/d BRV groups during treatment were 32.0, 44.2, and 55.8% separately (36). In another phase IIb study (N01114; NCT00175929), the responder rate of auxiliary BRV treatment is 30.8–39.6% (37). Phase III experiment (NO: 01253; NCT00464269) using fixed dose scheme found that the responder rate of BRV (5, 20, 50 mg/d) after treatment was 32.7% (38). In phase III study (NO: 1358; NCT01261325), the responder rate of 100 mg/d, 200 mg/d BRV group were 38.9, 37.8%, respectively, (39). Our meta-analysis showed that the response rate of epileptic seizure reduction ≥50% in the random effects model was 35%, which was generally consistent with the response rate of adult POS treated with adjuvant BRV.

In addition, in view of the long-term and extensive impact of childhood seizures, the aim of epilepsy treatment is to eliminate seizures (40). Studies have proved that early realization of epilepsy control and seizure freedom is conducive to early surgical intervention during long-term absence of seizures or cessation of ASM, which may lead to good cognitive development results (41). In our meta-analysis, we found that the rate of seizure freedom (no seizures) in children was 18%, which was higher than that in adults. Although the possibility of epileptic seizure freedom in children after BRV is not very significant, it is a treatment that can help reduce the burden of epilepsy in children and their families.

The adverse reactions of ASM can help us predict the quality of life of patients receiving ASM as a way to relieve and control seizures, but they have nothing to do with the final outcome of seizures. It is undeniable that children are more likely than adults to be affected by the adverse effects of ASM, and the toxic effects may also be different (34). Children treated with new ASM have various adverse reactions: lethargy, accidental injury, vomiting and adverse behavior events (aggression, hostility, hyperactivity) caused by levetiracetam. The incidence of these adverse reactions is higher in children than in adults (7). Topiramate seems to affect attention and language functions (5). Zonisamide can lead to loss of appetite, weight loss, lethargy, etc., and has a higher risk of mental disorders (10). The hepatotoxicity caused by sodium valproate is very typical in children, and it shows more bad behavior than ASM except for LEV (42, 43), and vomiting, somnolence, dizziness, etc. in the treatment with Oxcarbazepine (9).

Common treatment-emergent adverse events such as sedation or somnolence, decreased appetite or increased appetite, irritability, and bad behavior (such as irritability, aggression, etc.) can also occur after the use of BRV as an adjuvant treatment or after the conversion from LEV to BRV. Among them, somnolence is the most common adverse event. The occurrence of adverse behavior events with BRV is less than that after LEV treatment. Epileptiform discharges during the interictal period of epilepsy may disrupt sleep homeostasis at the local or systemic level. In addition, antiepileptic drugs may cause adverse reactions such as sleep disorders in patients with epilepsy (44). Our systematic review and meta-analysis showed that children with epilepsy have relatively good tolerance to BRV treatment. The incidence rate of TEAE in the 9 studies was 39%, most of which were mild to moderate. Somnolence was with an occurrence rate of 9% and mental disorders or bad behavior occurred in 12 percent of cases Remarkably, the incidence of severe TEAE was extremely low, and the deaths were not considered to be caused by BRV treatment. These safety results are consistent with the safety of adult patients receiving BRV treatment. Most adverse events (such as drowsiness, sedation and fatigue) will improve with the decrease of dosage, which can be solved by fractional administration or controlled release agent to increase tolerance (34). Therefore, most of the TEAE of BRV, in the treatment of childhood epilepsy, can be alleviated after adjustment to increase tolerance. Of course, this part of evidence needs to be further tested and studied.

This study has some limitations. Firstly, data on secondary outcomes were unavailable or missing in some studies. Secondly, although this study supports the effectiveness and safety of BRV in the treatment of epilepsy of all seizure types in children, it does not involve separate studies on specific epilepsy types (such as focal epilepsy, generalized epilepsy and epilepsy of unknown etiology according to ILAE) (45). Thirdly, due to the limitations of ASM approved for the treatment of epilepsy in children, our study only analyzed single-arm studies and retrospective studies. We look forward to more large sample multi-center clinical trials being published in the future. Fourthly, because of the differences in participants’ age, race, epileptic type, cause of seizures, severity of seizures, administration type and dose, combination of other ASMs, follow-up time, evaluation criteria, etc., the heterogeneity included in the study was significant. Fifthly, although we have conducted a comprehensive search, the number of articles that finally meet the standards was still limited. The reasons for the high heterogeneity of some indicators could not be further investigated by subgroup analysis or meta regression, and the risk of bias was inevitable. In addition, when it is difficult to assess subjective symptoms, especially due to children’s poor expression ability, clinical detection of toxic effects (such as pharmacokinetics monitoring, ASM serum concentration detection, and EEG monitoring) can reduce the side effects of ASM effectively (46). Because of the limited number of literatures that were able to extract relevant data of toxic effects, the plan to carry out this assessment was unsuccessful.

## 5. Conclusion

The systematic review and meta-analysis has proved that BRV is effective and safe in the treatment of childhood epilepsy. Compared with levetiracetam, it has no significant difference in efficacy, and even fewer adverse reactions. Of course, the determination of efficacy and safety of BRV has a greater clinical application prospect for children with drug resistance after long-term use of LEV, to treat epilepsy. The study on BRV treatment for children with epilepsy requires to expand the sample size validation. Similarly, the efficacy, safety and whether BRV is superior to other ASMs, still needs more clinical trial evidence. We expect that new randomized controlled trials will be carried out in the future to bring forward further validation.

## Data availability statement

The original contributions presented in the study are included in the article/Supplementary material, further inquiries can be directed to the corresponding author.

## Author contributions

TS, LF, and YX: data curation, methodology, and formal analysis. MP, JG, and XZ: software. YW: funding acquisition. TS: writing—original draft. YW: writing—review and editing. TS, LF, YX, MP, JG, XZ, and YW: conceptualization. All authors commented on previous versions of the manuscript, read and approved the final manuscript.

## Funding

This work was supported by Yuandu Scholars and Weifang Key Laboratory, and the Clinical Research Center of Affiliated Hospital of Weifang Medical University (No. 2022WYFYLCYJ02).

## Conflict of interest

The authors declare that the research was conducted in the absence of any commercial or financial relationships that could be construed as a potential conflict of interest.

## Publisher’s note

All claims expressed in this article are solely those of the authors and do not necessarily represent those of their affiliated organizations, or those of the publisher, the editors and the reviewers. Any product that may be evaluated in this article, or claim that may be made by its manufacturer, is not guaranteed or endorsed by the publisher.
