# Supplementary material for: Safety and efficacy of brivaracetam in children epilepsy: a systematic review and meta-analysis
Source: Front Neurol. 2023 Jul 6;14:1170780. doi: 10.3389/fneur.2023.1170780 (PMC10359931; doi:10.3389/fneur.2023.1170780)
Supplement: Supplementary file 1 [file Table_1.docx]

Supplementary Material

Safety and efficacy of brivaracetam in children epilepsy: A systematic review and meta-analysis

Ting Song^1^, Lingjun Feng^2^, Yulei Xia^1^, Meng Pang^1^, Jianhong Geng^1^, Xiaojun Zhang^1^ and Yanqiang Wang^1^*

*** Correspondence:** Yanqiang Wang: wangqiangdoctor@126.com

# Supplementary Figures and Tables

## Supplementary Tables

**Supplementary Table 1.** Literature retrieval strategy of the electronic databases.

**Search Strategy in PubMed**

| Search number | Query | Results |
| --- | --- | --- |
| 5 | (#1 OR #2) AND (#3 OR #4) | 300 |
| 4 | "epilepsy"[Title/Abstract] OR "acute epilepsy"[Title/Abstract] OR "Awakening Epilepsy"[Title/Abstract] OR "chronic epilepsy"[Title/Abstract] OR "Cryptogenic Epileps*"[Title/Abstract] OR "epilepsi*"[Title/Abstract] OR "epileptic"[Title/Abstract] OR "epileptic disorder"[Title/Abstract] OR "epileptic syndrome*"[Title/Abstract] OR "falling sickness"[Title/Abstract] OR "seizure disorder*"[Title/Abstract] OR "tardy epilepsy"[Title/Abstract] | 145,722 |
| 3 | "Epilepsy"[Mesh] | 122,297 |
| 2 | "Brivaracetam"[Title/Abstract] OR "Briviact"[Title/Abstract] OR "brivlera"[Title/Abstract] OR "nubriveo"[Title/Abstract] OR "rikelta"[Title/Abstract] OR "UCB 34714"[Title/Abstract] OR "UCB34714"[Title/Abstract] | 378 |
| 1 | "brivaracetam" [Supplementary Concept] | 228 |

**Search Strategy in Web of Science**

# Web of Science Search Strategy (v0.1)

# Database: All Databases

# Entitlements:

- WOS: 1900 to 2022

- CSCD: 1989 to 2022

- DIIDW: 1966 to 2022

- KJD: 1980 to 2022

- MEDLINE: 1950 to 2022

- SCIELO: 2002 to 2022

#Date run:Wed Aug 10 2022

# Searches:

| Search number | Query | Results |
| --- | --- | --- |
| #94 | Brivaracetam (Topic) OR Brivaracetam (Title) OR Briviact (Title) OR brivlera (Title) OR nubriveo (Title) OR rikelta (Title) OR UCB 34714 (Title) OR UCB34714 (Title) | 810 |
| #98 | epilepsy (Topic) OR epilepsy (Title) OR acute epilepsy (Title) OR Awakening Epilepsy (Title) OR chronic epilepsy (Title) OR Cryptogenic Epileps* (Title) OR epilepsi* (Title) OR epileptic (Title) OR epileptic disorder (Title) OR epileptic syndrome* (Title) OR falling sickness (Title) OR seizure disorder* (Title) OR tardy epileps (Title) | 274532 |
| #99 | #94 AND #98 | 535 |

**Search Strategy in Embase**

Date: 2022/8/10

| No. | Query | Results |
| --- | --- | --- |
| #5 | (#1 OR #2) AND (#3 OR #4) | 925 |
| #4 | 'epilepsy'/exp | 280249 |
| #3 | 'epilepsy':ti,ab,kw OR 'acute epilepsy':ti,ab,kw OR 'awakening epilepsy':ti,ab,kw OR 'chronic epilepsy':ti,ab,kw OR 'cryptogenic epileps*':ti,ab,kw OR 'epilepsi*':ti,ab,kw OR 'epileptic':ti,ab,kw OR 'epileptic disorder':ti,ab,kw OR 'epileptic syndrome*':ti,ab,kw OR 'falling sickness':ti,ab,kw OR 'seizure disorder*':ti,ab,kw OR 'tardy epilepsy':ti,ab,kw | 216210 |
| #2 | 'brivaracetam'/exp | 1056 |
| #1 | 'brivaracetam':ti,ab,kw OR 'briviact':ti,ab,kw OR 'brivlera':ti,ab,kw OR 'nubriveo':ti,ab,kw OR 'rikelta':ti,ab,kw OR 'ucb 34714':ti,ab,kw OR 'ucb34714':ti,ab,kw | 632 |

**Search Strategy in Cochrane**

Date Run: 10/08/2022 00:21:38

| ID | Search | Results |
| --- | --- | --- |
| #1 | (‘Brivaracetam’ OR ‘Briviact’ OR ‘brivlera’ OR ‘nubriveo’ OR ‘rikelta’ OR ‘UCB 34714’ OR ‘UCB34714’):ti,ab,kw | 153 |
| #2 | (‘epilepsy’ OR ‘acute epilepsy’ OR ‘Awakening Epilepsy’ OR ‘chronic epilepsy’ OR ‘Cryptogenic Epileps*’ OR ‘epilepsi*’ OR ‘epileptic’ OR ‘epileptic disorder’ OR ‘epileptic syndrome*’ OR ‘falling sickness’ OR ‘seizure disorder*’ OR ‘tardy epilepsy’):ti,ab,kw | 9628 |
| #3 | MeSH descriptor: [Epilepsy] explode all trees | 2598 |
| #4 | #1 AND (#2 OR #3) | 124 |

**Supplementary Table 2.** The previous antiseizure medications and concomitant ASMs

| **Study(author/year)** | **N** | **Number of prior ASMs** | **Number of concomitant ASMs** | **Type of concomitant ASMs, n (%)** |
| --- | --- | --- | --- | --- |
| Schubert-Bast,2018 | 34 | 1.7± 2.2 | 1.6±0.7 | Levetiracetam, 20 (59), Laotrigine,12(35), Lacosamide,9(27) Oxcarbazepine,8(24), Valproate,6(18), Carbamazepine, 4(12) |
| Liu,2019 | 99 | 0–1 37(37.4%) 2–4 32(32.3%) 5 or more 30(30.3%) | 1 32(32.3%)  2 41(41.4%) 3 or more 26(26.3%) | Valproate, 51 (51.5), Topiramate, 27 (27.3), Lamotrigine, 17 (17.2) Clobazam, 14 (14.1), Phenobarbital, 14 (14.1), Oxcarbazepine, 13 (13.1) |
| Nissenkorn,2019 | 31 | Responders,8.5 ± 4.7 14(45.2%) Nonresponders,9.5 ± 4.4 17(54.8%) | 2.1 ± 1.25 | Levetiracetam etc. |
| McGuire,2019 | 20 | NA | Responders, 1.74 8(40%)  Nonresponders,2.5 12(60%) | Levetiracetam etc. |
| Patel,2019 | 149 | 3.6± 2.9 | 2.1 ± 1.0 | Valproate, 68 (45.6), Diazepam, 39 (26.2), Lamotrigine, 39 (26.2) Carbamazepine, 39 (26.2), Topiramate, 34 (22.8), Clobazam, 33 (22.1) Oxcarbazepine, 33 (22.1), Lacosamide,19 (12.8), Clonazepam, 15 (10.1) |
| Visa-Reñé,2020 | 46 | 1 4(8.7%) 2-3 12(26.1%) 4-5 10(21.7%) 6 or more 20(43.5%) | 1 22(47.8%) 2 12 (26.1%) ≥ 3 12 (26.1%) | Clobazam, 20 (43.5), Valproate, 19 (41), Oxcarbazepine, 7(15.2) Levetiracetam, 6(3), Lamotrigine, 5(10.8) |
| Ferragut,2021 | 66 | 1 4(6.6 %) 2 4(6.1%) 3 10(15.1 %) >4 48(72.3 %) | 0 5.5% 1 20% 2 40% 3 25 % >4 9.5 % | Levetiracetam etc. |
| Russo,2021 | 8 | 10± 3.3 | 2.3 | NA |
| Farkas,2022 | 50 | NA | NA | NA |

**Supplementary Table 3.** The type, semiology and etiology of childhood epilepsy

| **Study(author/year)** | **N** | **Type of epilepsy, n (%)** | **Seizure semiology, n (%)** | **Etiology of the epilepsy, n (%)** |
| --- | --- | --- | --- | --- |
| Schubert-Bast,2018 | 34 | Focal seizures, 34(100) | Focal onset seizures with preserved awareness, 18(52.9) Focal onset seizures with impaired awareness, 24(70.6) Focal to bilateral tonic–clonic seizures, 17 (50) Other, 9(26.5) | Structural, 41.2 (14) -Dysplasia, 11.8(4) -Neoplasia, 14.7 (5) -Postischemic, 9.0 (3) -Other, 5.9 (2) Genetic, 2.9 (1)  Unknown, 55.9 (19) |
| Liu,2019 | 99 | Focal seizures, 52(52.5) Primary generalized seizures,47(47.5) | Focal, 66(66.7) Generalized, 47(47.5) Unclassified, 6 (6.1) | NA |
| Nissenkorn,2019 | 31 | Focal seizures, 20(64.5)  Epileptic syndromes, 11(35.5)  -Lennox–Gastaut syndrome, 5(16.1) -Absence with eyelid myoclonus (Jeavons syndrome), 3(9.7) -Myoclonic–atonic epilepsy, 3(9.7) | Focal onset with impaired awareness,17(54.8) Drop attacks, 7(22.6) Myoclonic absence, 3(9.7) Focal to bilateral tonic–clonic, 2(6.5) Focal with preserved awareness, 2(6.5) | Abnormal MRI findings, 12(38.7) -Focal dysplasia 5(16.1) -Tumors 3(9.7) -Destructive lesions, 3 (9.7) Psychiatric comorbidity, 19(61.3): -Autistic spectrum disorder, 6 (19.4) -Intellectual disability, 5 (16.1) -Behavior disorder, 2 (6.5) |
| McGuire,2019 | 20 | Focal epilepsy，11(55) Generalized epilepsy, 6(30) Mixed epilepsy, 3(15) | Focal epilepsy，11(55)  Generalized epilepsy, 6(30) Mixed epilepsy, 3(15) | NA |
| Patel,2019 | 149 | Focal epilepsy, 149 (100) | Focal, 149 (100) Generalised,1 (0.7) Unclassified, 1 (0.7) | NA |
| Visa-Reñé,2020 | 46 | Epileptic encephalopathy, 18 (39.1) Focal epilepsy, 24 (52.1) Other, 4 (8.6): -Epilepsy with generalized tonic–clonic seizures, 2 (4.3) -Childhood absence epilepsy, 2 (4.3) | Generalised,20 (39.1) Focal with loss of consciousness, 15 (32.6) Focal without loss of consciousness, 14 (30.4) Focal with secondary generalization, 8 (17.4) | Structural 18 (39.1) -Perinatal hypoxia, 5(10.9) -Cortical dysplasia, 5(10.9) -Vascular causes, 5(10.9) -Trauma, 1(2.2) -Tumor, 1(2.2) -Brain infection, 1(2.2) Unknown 18 (39.1) Genetic 9 (19.6) Autoimmune encephalitis 1 (2.2) |
| Ferragut,2021 | 66 | Generalized epilepsy, 27(40.9) Focal epilepsy, 19(28.8) Multifocal epilepsy, 20(30.2) | Focal, 26(39.4) Generalized, 13(19.7) Epileptic encephalopathy, 27(40.9) | Focal, 26(39.4) Generalized, 13(19.7) Epileptic encephalopathy, 27(40.9): -Unclassifiable, 17(25.8) -Lennox-Gastaut syndrome, 5(7.6) -Continuous spike and wave during sleep syndrome, 3(4.5) -Epileptic spasm, 2(3.0) |
| Russo,2021 | 8 | Focal epilepsy, 4(50) Epileptic encephalopathy, 4(50) | Focal, 4 (50) Generalized, 4 (50) | Structural, 5 (62.5) Genetic, 3 (37.5) |
| Farkas,2022 | 50 | NA | NA | NA |
